# Supplementary material for: Data-driven analysis of motor activity implicates 5-HT2A neurons in backward locomotion of larval Drosophila
Source: Sci Rep. 2018 Jul 9;8:10307. doi: 10.1038/s41598-018-28680-8 (PMC6037780; doi:10.1038/s41598-018-28680-8)
Supplement: Supplementary file 1 — Supplementary Information [file 41598_2018_28680_MOESM1_ESM.pdf]

Data-driven analysis of motor activity implicates 5-HT2A neurons in backward locomotion of larval *Drosophila*

Jeonghyuk Park[1], Shu Kondo[3], Hiromu Tanimoto[4], Hiroshi Kohsaka[2], and Akinao Nose[1,2,*]

[1]Department of Physics, Graduate School of Science, University of Tokyo, Tokyo 113-0033, Japan
[2]Department of Complexity Science and Engineering, University of Tokyo, Chiba 277-8561, Japan
[3]Invertebrate Genetics Laboratory, National Institute of Genetics, Mishima, Shizuoka 411-8540, Japan
[4]Graduate School of Life Sciences, Tohoku University, Sendai 980-8577, Japan
[*]nose@k.u-tokyo.ac.jp

Supplementary Figure S1-7
Supplementary Video S1-8

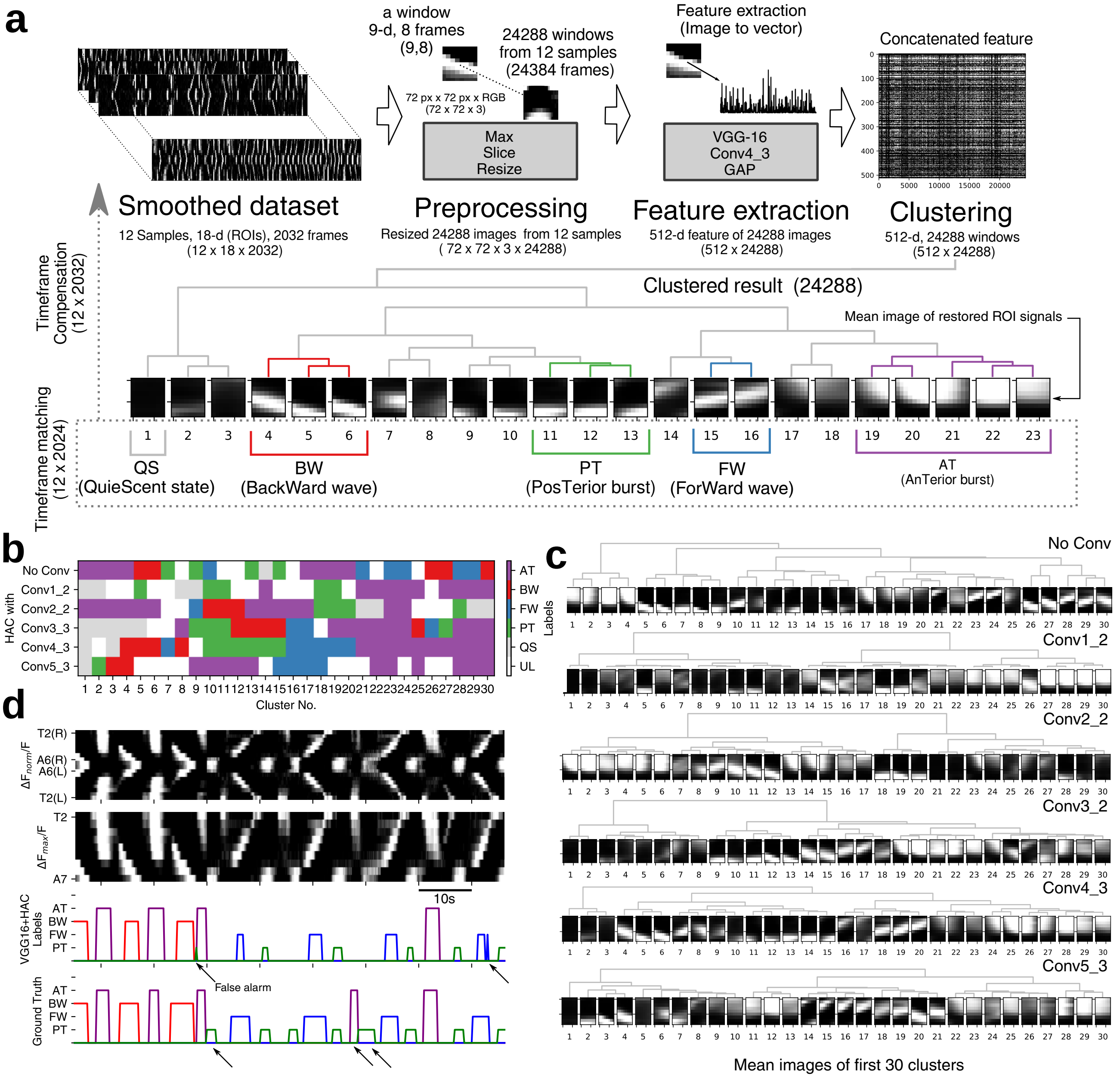

Figure S1. related to Fig. 1 and Methods. (a) Details of feature extraction and clustering. (b, c) Evaluation of cluster analysis using features from different layers of VGG16. Cluster analyses of the five convolution layers of VGG16 (Conv1_2, Conv2_2, Conv3_2, Conv4_3 and Conv5_3) and the original data. Note that input of HAC must be 1-d vector, windows are flattened to vectors for HAC without convolution layer (No Conv). Each cluster was manually inspected to determine if it represents a motor pattern. Conv4_3+HAC gave the best performance among the layers in that it identified all four motor patterns (AT, PT, FW and BW) and in a well-separated manner. (d) An example of comparison between the original activity data (top, 18-d ROI and 9-d ROI images), motor patterns assigned by VGG16+HAC (VGG16+HAC labels) and human inspection (ground truth). Visual cues indicate false alarms.

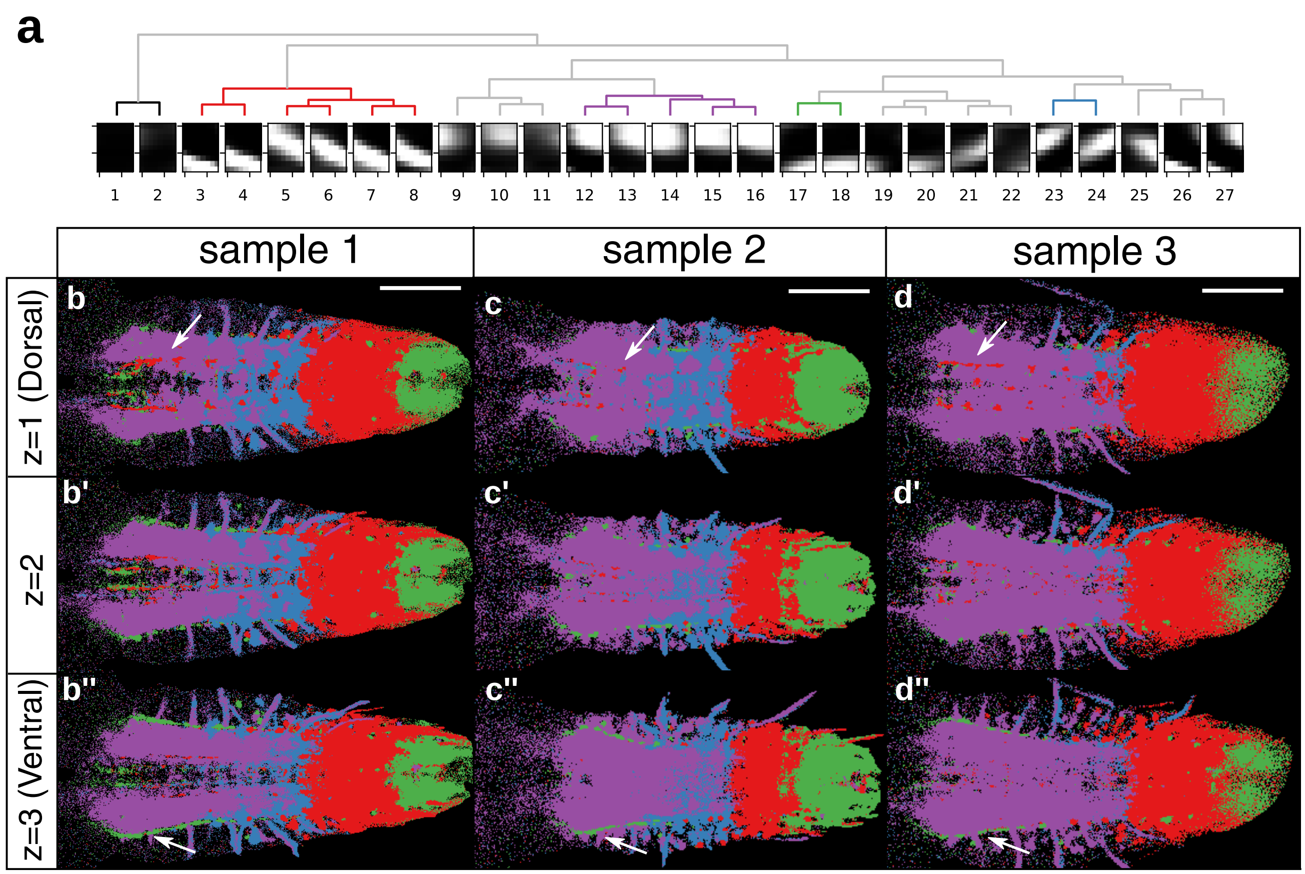

Figure S2. related to Fig. 3. (a) Clustering analyses of the Conv4_3 of VGG16 derived from activity data of all neurons. (b-d") Dominant motor pattern mapping. Same as Fig. 3c but voxels are colored only for the most dominant motor pattern (highest Pearson's r). Three z sections from dorsal to ventral are shown. Visual cues indicate structures specifically active during BW and PT. Note that b-b" is derived from the same sample as Fig. 3c. Scale bar 100μm

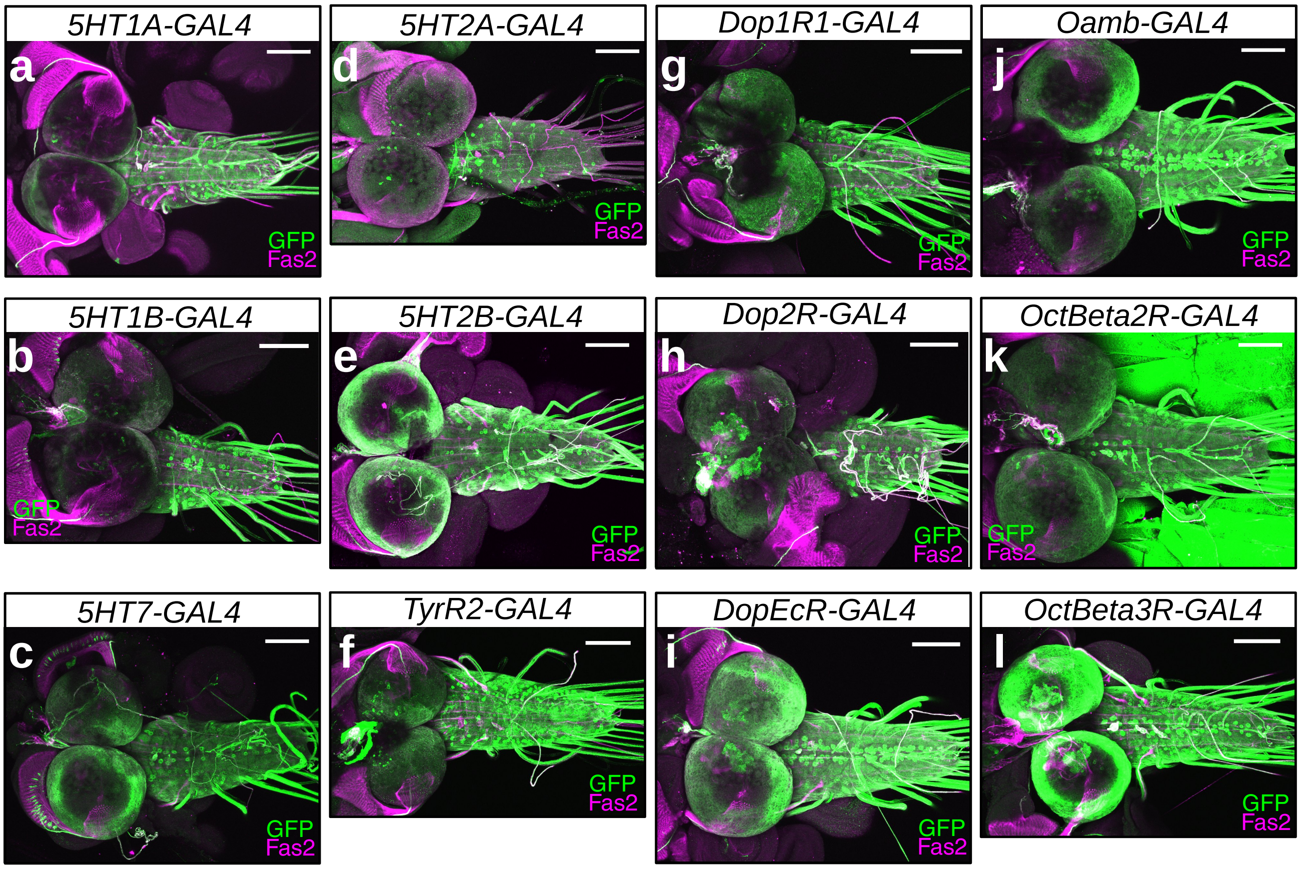

Figure S3. Expression of neuromodulator receptor GAL4 lines. related to Fig. 4. Fas2 (magenta) was used as a reference marker. *5-HT1A* (a), *5-HT1B* (b), *5-HT7* (c), *5-HT2A* (d), *5-HT2B* (e), *TyrR2* (f), *Dop1R1* (g), *Dop2R* (h), *DopEcR* (i), *Oamb* (j), *OctBeta2R* (k), *OctBeta3R* (l). Scale bar 100μm.

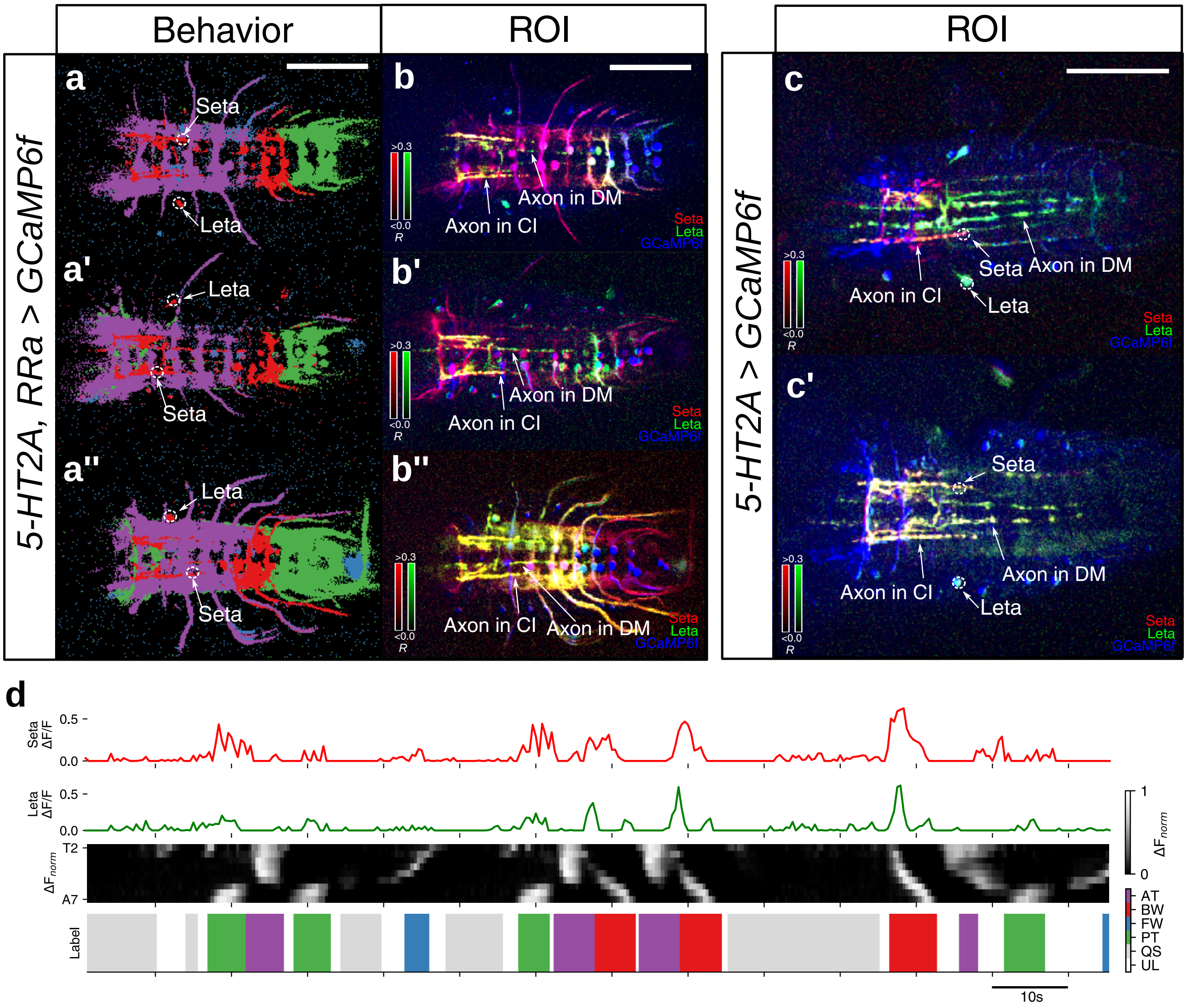

Figure S4. related to Fig. 4. (a-c) Dominant motor mapping (a-a”) and ROI correlation mapping (b-b”) as in Fig. 4c, and ROI correlation mapping (c, c’) as in Fig. 4f”, showing more samples. Note that a, b and c are the same as Fig. 4c’, C” and f”, respectively. (d) Activity of Seta and Leta neurons. Activity of single Seta (top, ΔF/F of Seta), and Leta (middle, ΔF/F of Leta) aligned with mapped motor patterns (bottom, ΔF_{norm} of MNs and label). Color code of motor pattern as in Fig. 2. Scale bar 100μm.

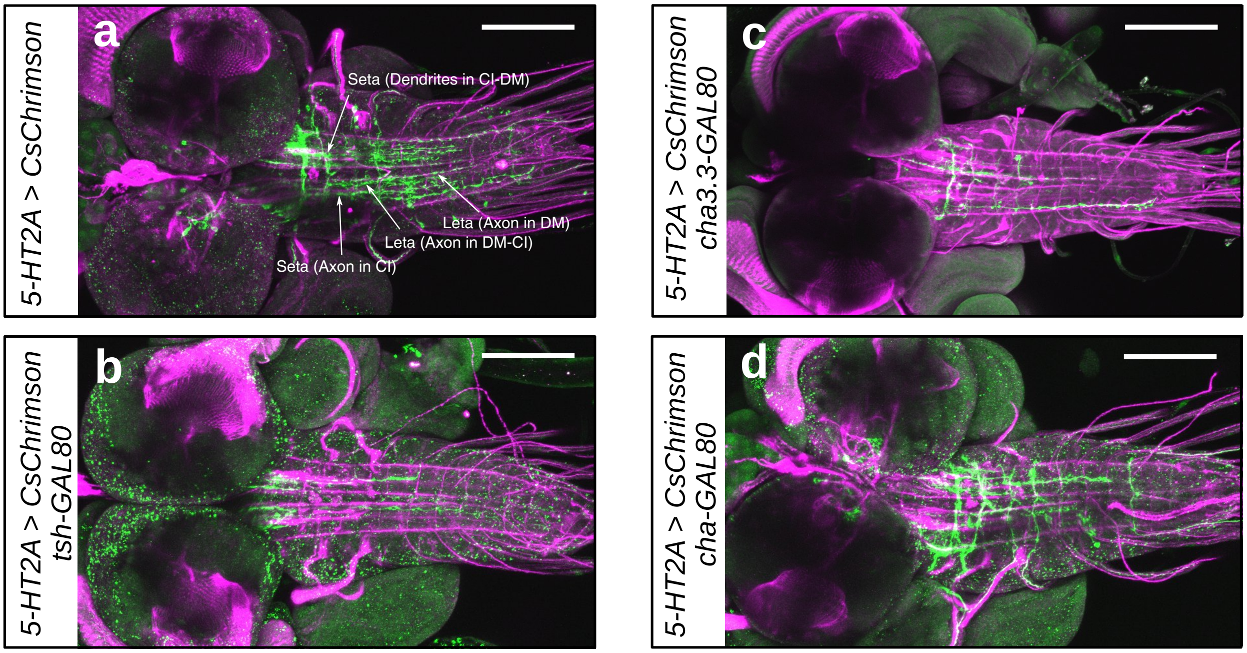

Figure S5. related to Fig. 6. Expression of CsChrimson driven by *5-HT2A-GAL4* (a) and its partial suppression by *tsh-GAL80* (b), *cha3.3-GAL80* (c) and *cha-GAL80* (d). Expression in Seta and Leta is absent in (b-d). Scale bar 100μm.

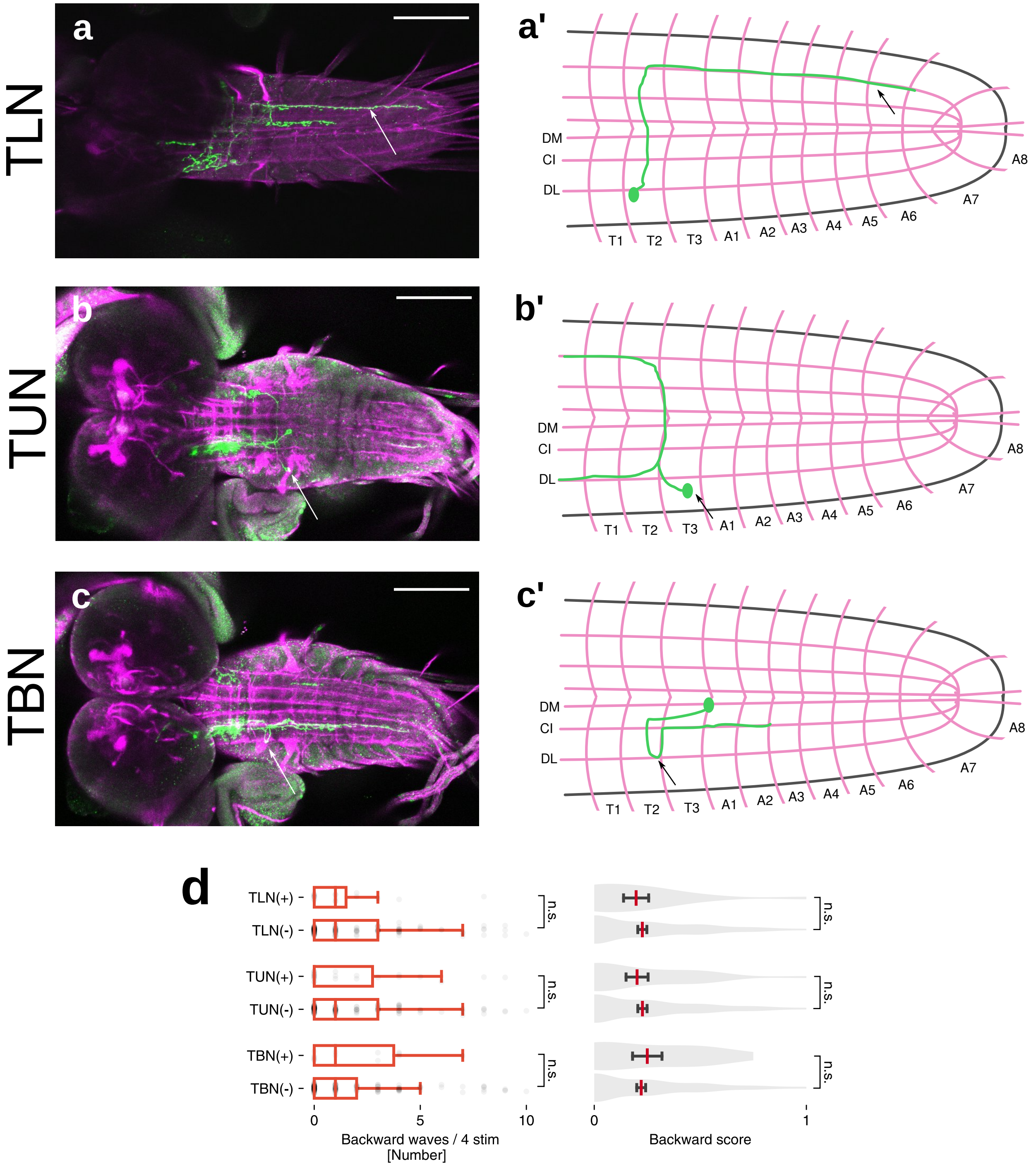

Figure S6. related to Fig. 6. Morphology of *5-HT2A-GAL4*-targeted neurons used as controls. (a-c) Immunostaining (green, a-c) and schematic diagram showing the morphology of TLN (a, a'), TUN (b, b') and TBN (c, c'). Fas2 landmarks are also shown in a-c (magenta). (d) Quantification of the number of backward peristalsis (left, boxplot with scatter) and backward score (right, violinplot with mean and SEM) in larvae with or without CsChrimson expression in TLN, TUN and TBN. p=0.35 (waves), 0.36 (score) for TLN (n=19 (+), 161 (-)), p=0.44 (waves), 0.32 (score) for TUN (n=26 (+), 154 (-)), p=0.24 (waves), 0.29 (score) for TBN (n=14 (+), 166 (-)). P-value is derived from Mann–Whitney *U* test. Scale bar 100μm.

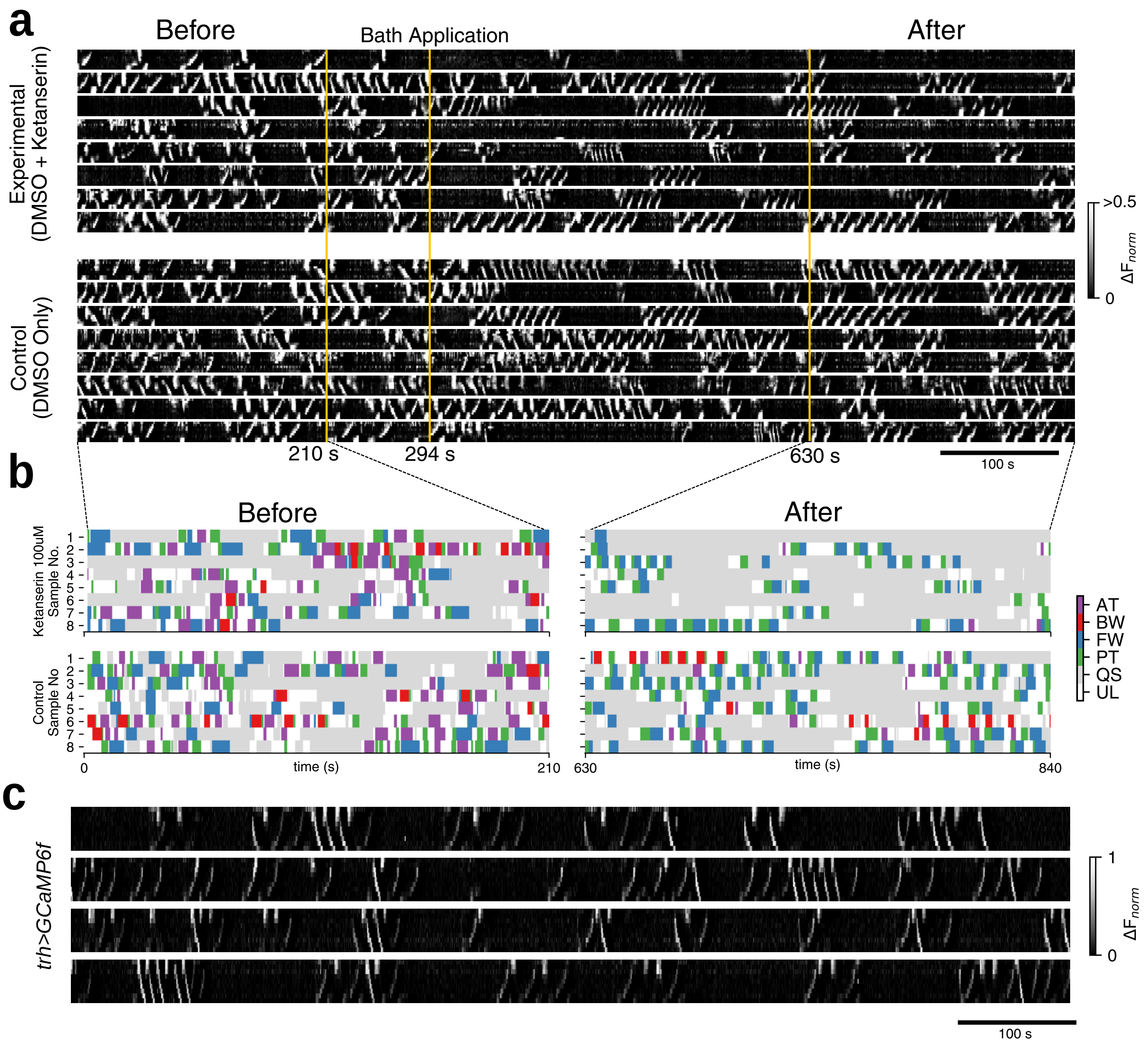

Figure S7. related to Fig. 7. (a-b) Segmental activity (T2-A7 from top to bottom in each row, ΔF_{norm}) (a) and extracted motor pattern (b) before and after the application of Ketanserin obtained from 16 *5-HT2A,RRa>GCaMP6f* larvae. (c) Serotonergic neurons show stronger activity during BW and AT compared to during FW and PT. Segmental activity pattern (T2-A7 from top to bottom in each row, ΔF_{norm}) from four *trh>GCaMP6f* larvae.

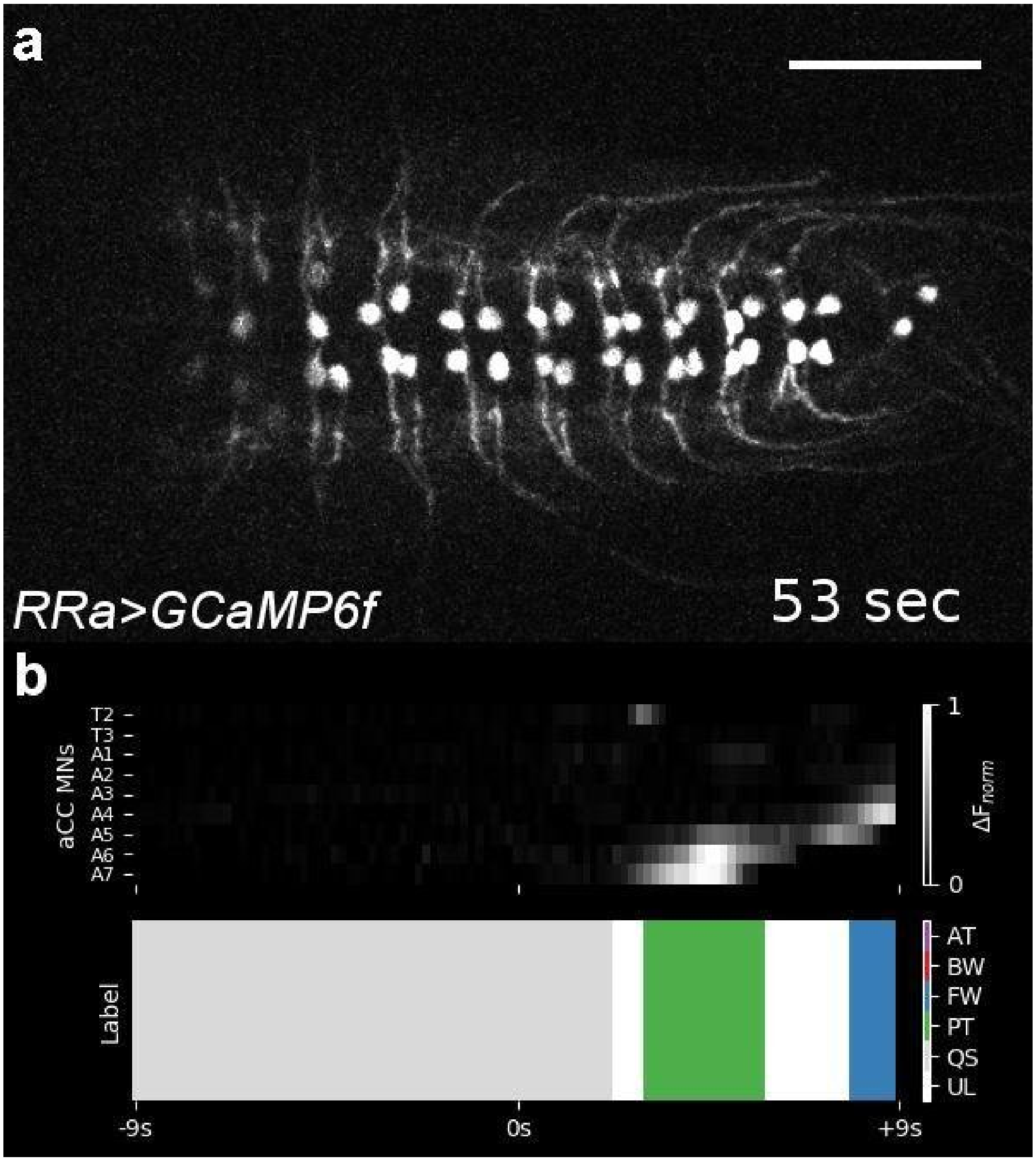

Video S1. related to Fig2. a', a"'. (a) Calcium imaging movie of a sample of *RRa>GCaMP6f* with 5.4x speed. (b) pre-processed ΔF_{norm} of 9-d timeseries displayed as a grayscale image (top) and label (bottom). Scale bar 100μm.

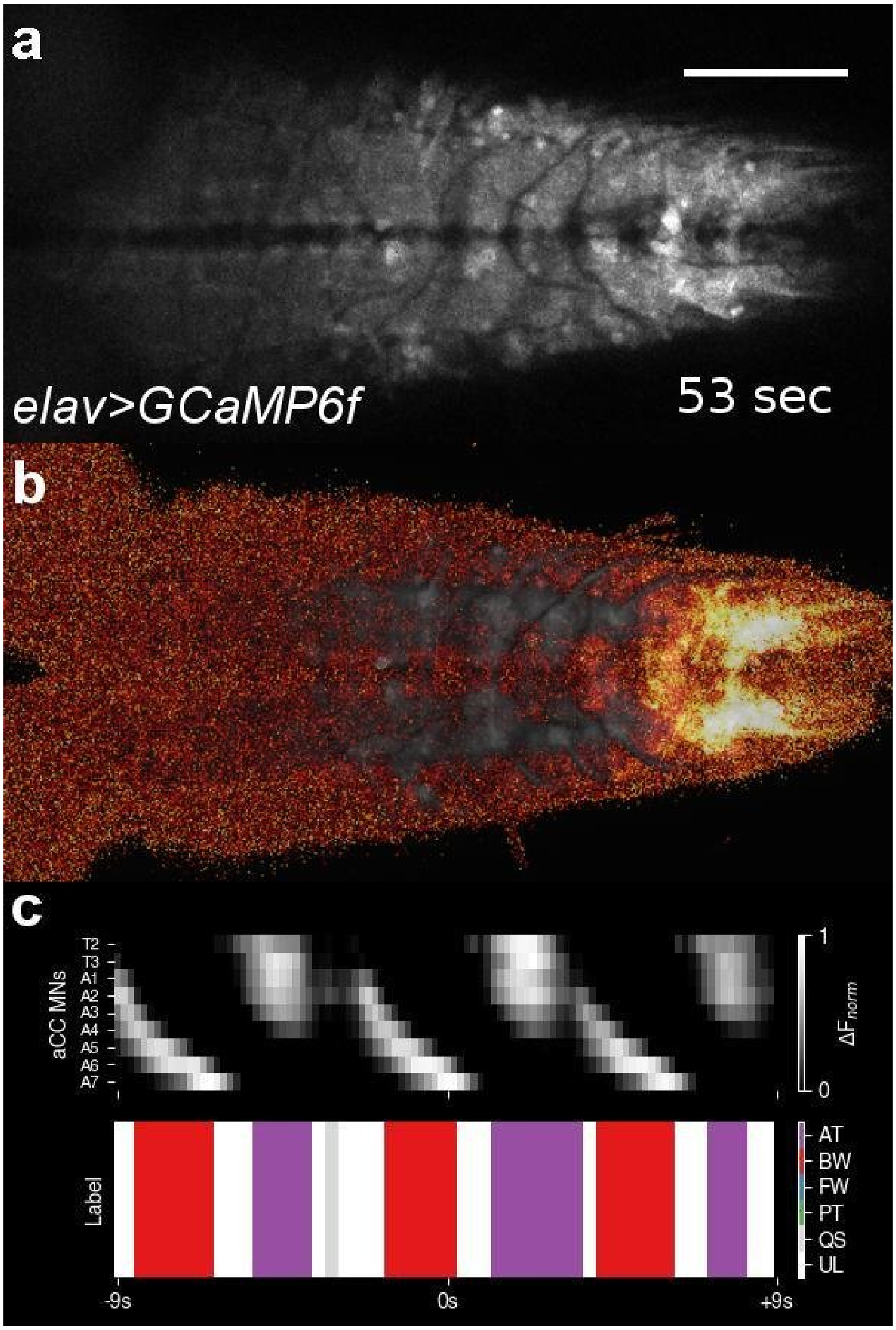

Video S2. related to Fig. 3a. (a) Calcium imaging movie of a sample of *elav>GCaMP6f* with 5.4x speed. (b) Voxel-wise smoothed movie of (a). (c) pre-processed ΔF_{norm} of 9-d timeseries displayed as a grayscale image (top) and label (bottom). Scale bar 100μm.

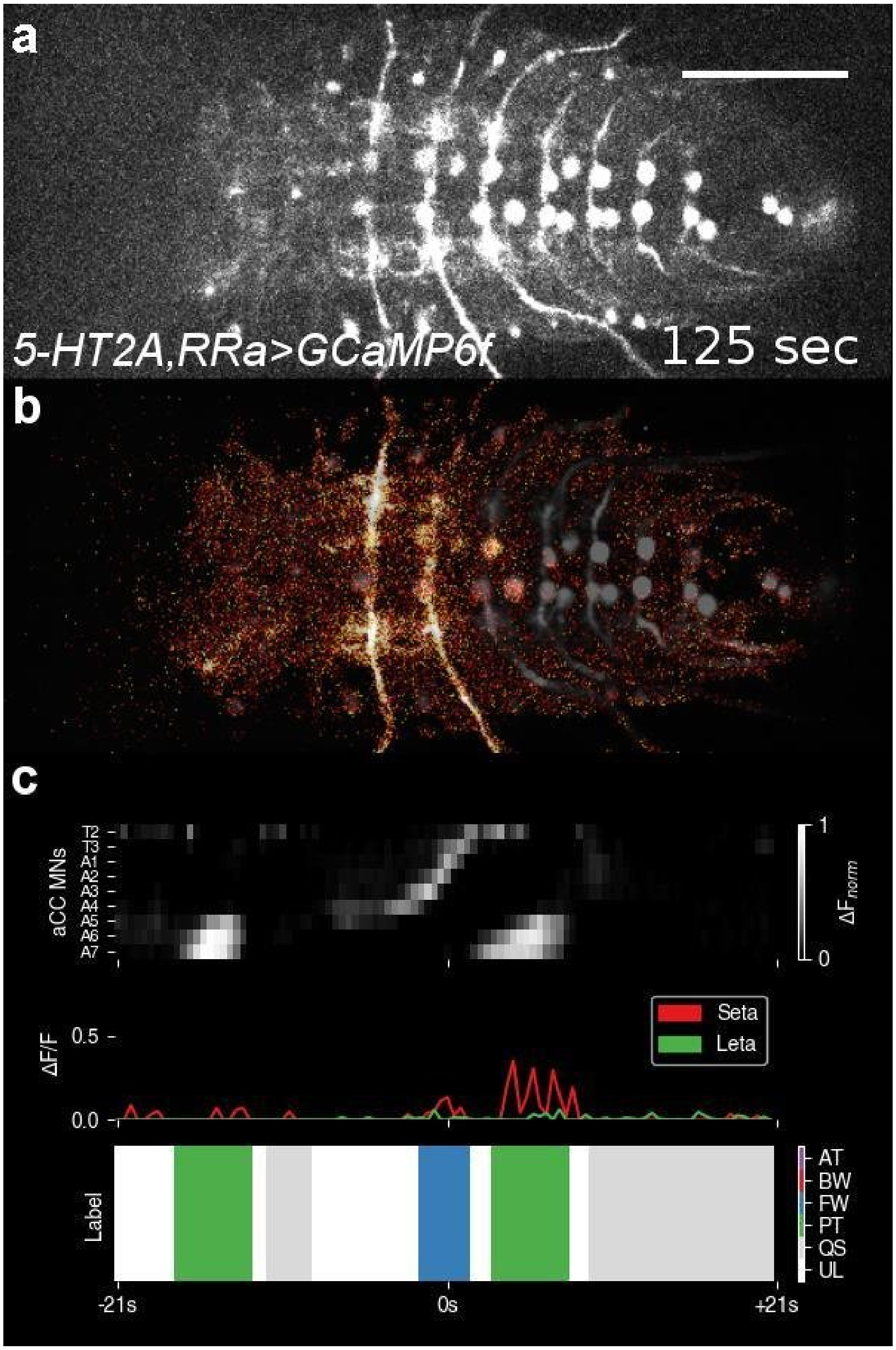

Video S3. related to Fig. 4a. (a) Calcium imaging movie of a sample of *5-HT2A, RRa>GCaMP6f* with 12.6x speed. (b) Voxel-wise smoothed movie of (a). (c) pre-processed ΔF_{norm} of 9-d timeseries displayed as a grayscale image (top), ΔF/F of Seta/Leta (middle) and label (bottom). Scale bar 100μm.

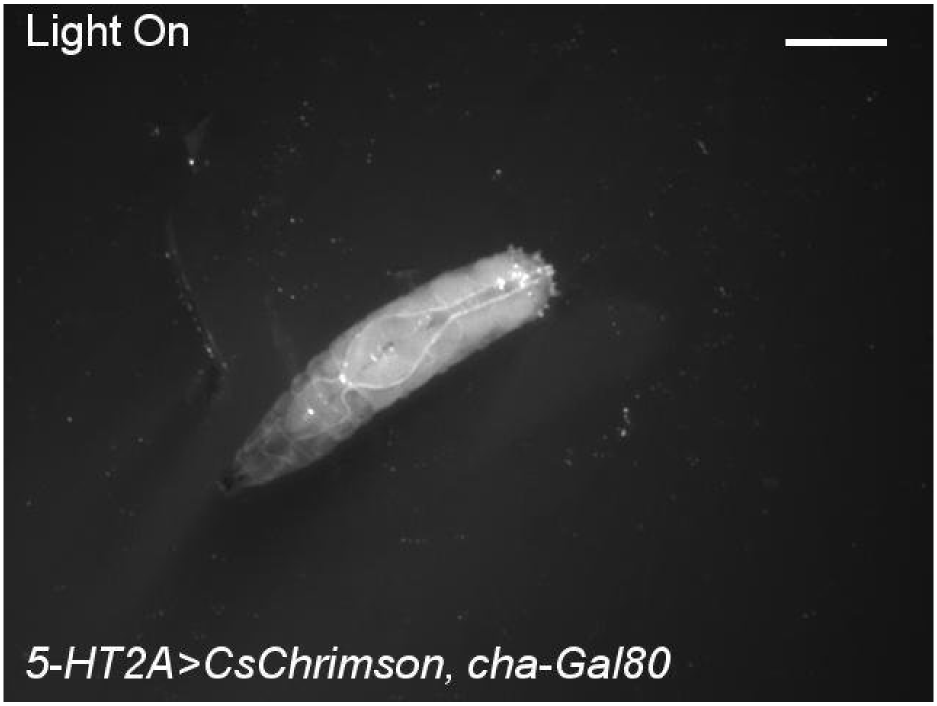

Video S4. related to Fig. 6b. Optogenetic activation of *5-HT2A>CsChrimson* and *5-HT2A>CsChrimson*. 2x speed. Scale bar 1mm.

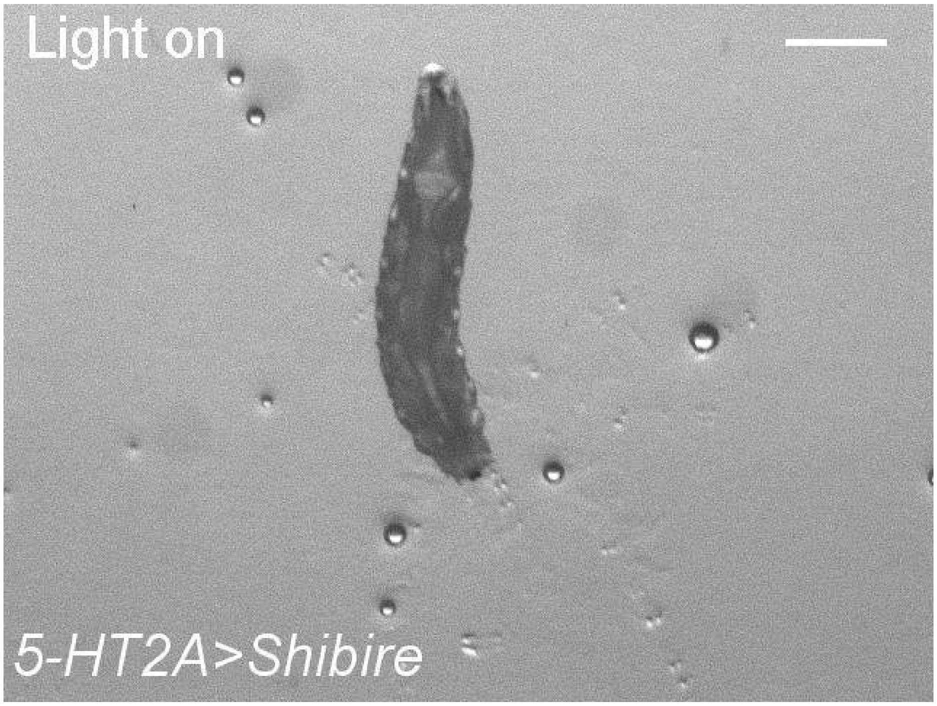

Video S5. related to Fig. 7a. Blue light stimulation of *UAS-Shibire*, *5-HT2A>Shibire*, 2x speed. Scale bar 1mm.

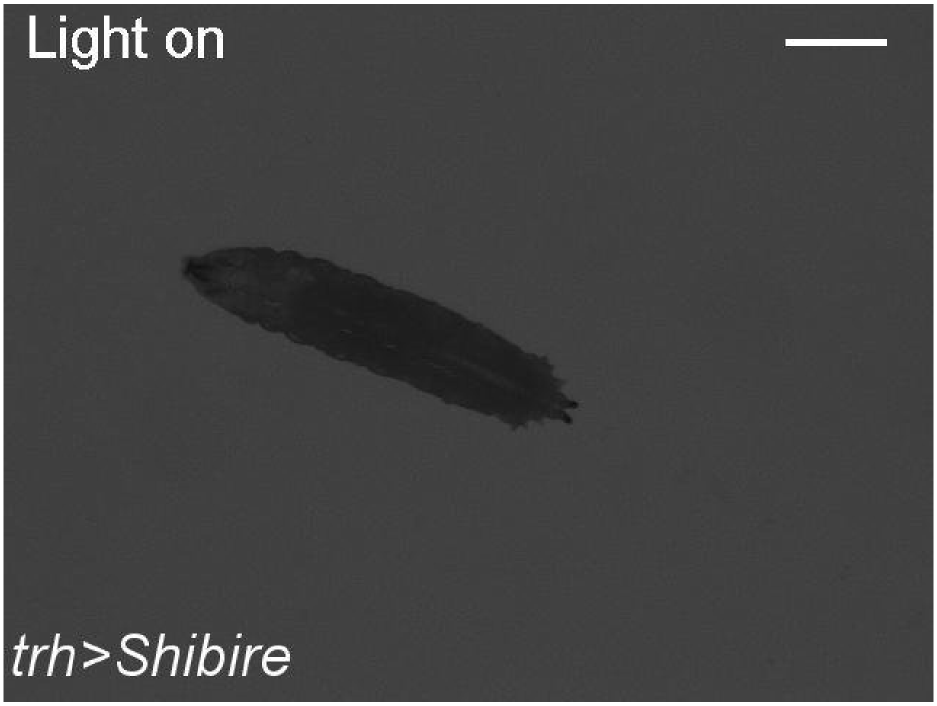

Video S6. related to Fig. 7a. Blue light stimulation of *trh-GAL4* and *trh>Shibire*. 2x speed. Scale bar 1mm.

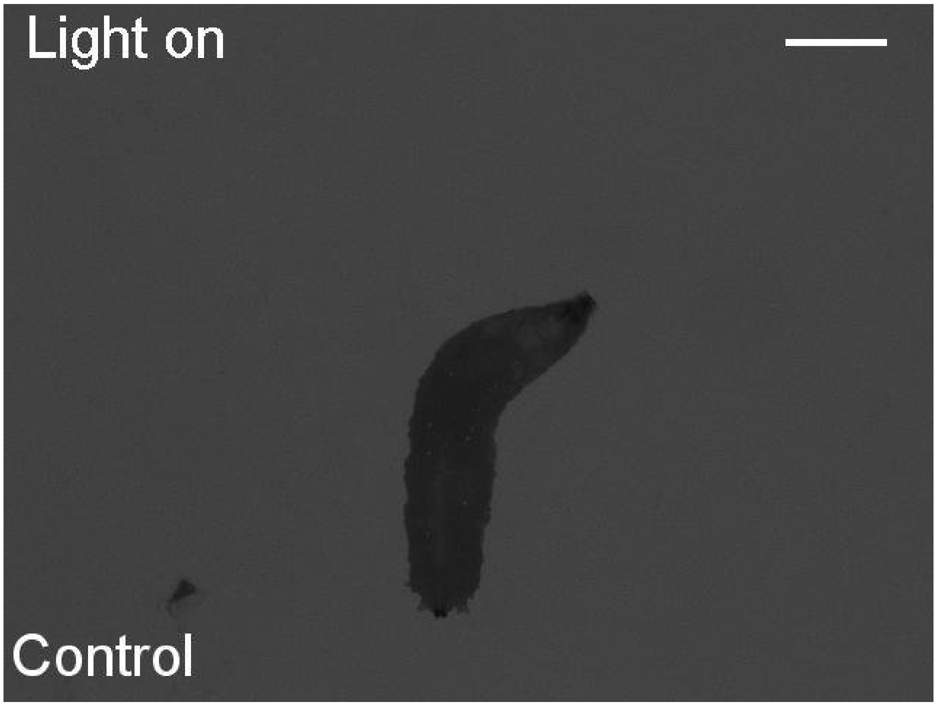

Video S7. related to Fig. 7b. Blue light stimulation of *5-HT2A*^{PL}. 2x speed. Scale bar 1mm.

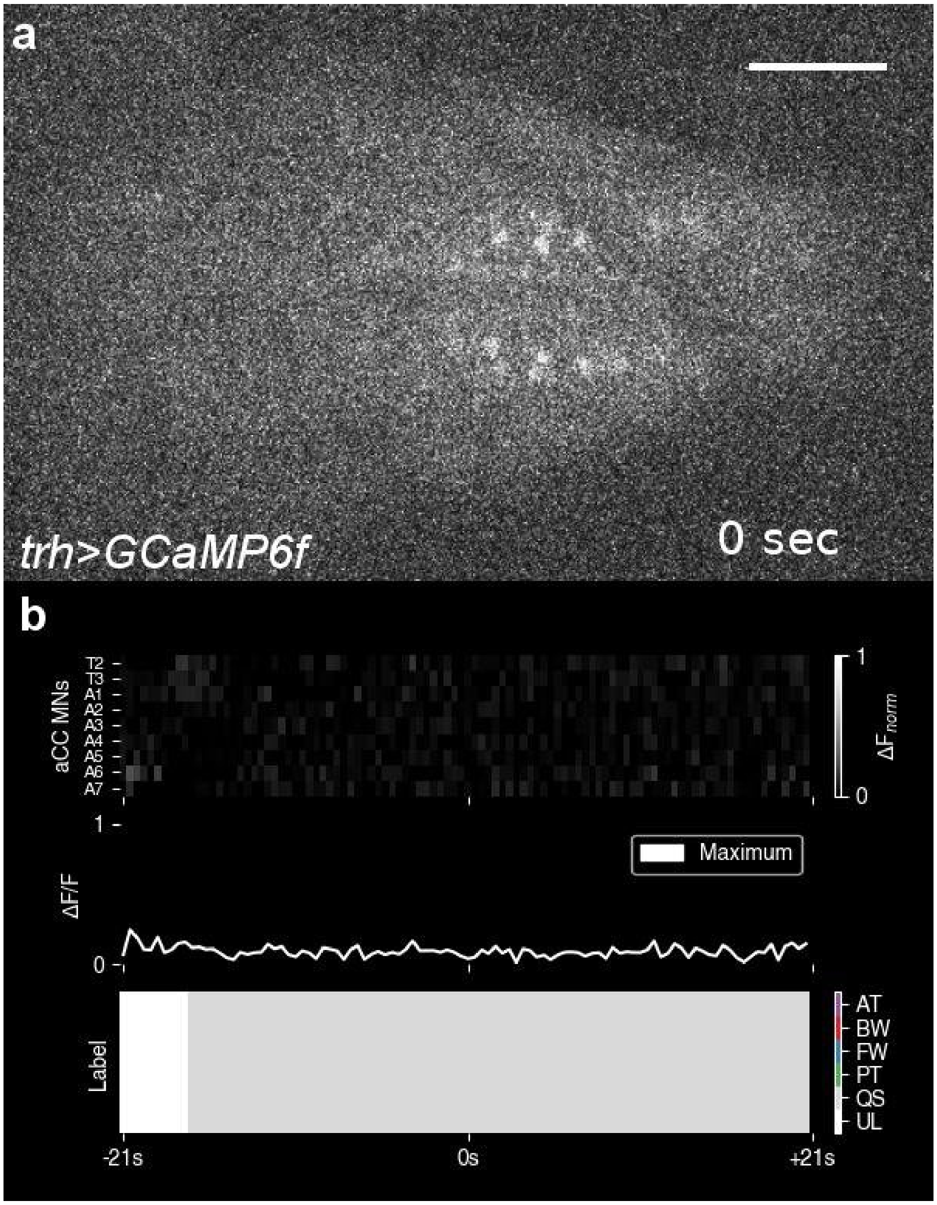

Video S8. related to Fig. 4a. (a) Calcium imaging movie of a sample of *trh>GCaMP6f* with 12.6x speed. (c) pre-processed ΔF_{norm} of 9-d timeseries displayed as a grayscale image (top), Maximum ΔF/F within ROIs (middle) and label (bottom). Scale bar 100μm.
